# The inter‐relationship between diet, selflessness, and disordered eating in Australian women

**DOI:** 10.1002/brb3.1774

**Published:** 2020-08-07

**Authors:** Melissa Collins, Stephanie Quinton

**Affiliations:** ^1^ Charles Sturt University Albury NSW Australia

**Keywords:** diet, disordered eating, perception of the self, selflessness, veganism, vegetarianism

## Abstract

Personality traits and diet can be used to predict if a person is predisposed to disordered eating. Results of this study demonstrate a strong significant relationship between the personality trait of selflessness, diet group, and disordered eating. Vegans were most likely to display selflessness tendencies associated with disordered eating; however when selflessness was controlled for, vegans displayed substantially less disordered eating pathology than non‐vegetarians.

**Objective:**

To explore the relationship between diet group (non‐vegetarian, semi‐vegetarian, true‐vegetarian, and vegan) and disordered eating while investigating to what extent personality trait of selflessness mediates the relationship between diet group and disordered eating.

**Method:**

Cross‐sectional data from 634 Australian nonclinical women who completed a series of online questionnaires including measures of diet group, disordered eating, and selflessness were used to examine associations between diet, personality (selflessness), and disordered eating.

**Results:**

Selflessness was found to be a significant positive predictor of disordered eating. Results confirm that selflessness played a suppressing role in the relationship between the vegan diet group and disordered eating, when compared to non‐vegetarians. Surprisingly, vegans displayed significantly less disordered eating than non‐vegetarians and semi‐vegetarians.

**Discussion:**

Results of the current study imply that the role of selflessness on disordered eating, when broken down across diet group, may be more complex than first thought. If replicated, these results suggest that targeted treatment of selflessness in different diet groups may improve treatment outcomes for disordered eating. Further research should explore why diet groups differ in terms of selflessness and how this impacts disordered eating.

## Graphical abstract

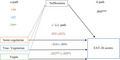

## 1 INTRODUCTION

Disordered eating is a significant issue in the Australian population, with an estimated 15% of Australian women needing clinical treatment for eating disorders (EDs) during their lifetime (National Eating Disorders Collaboration, 2012). Disordered eating results from dysfunctional feelings, behaviors, and thoughts concerning eating, feeding, and body image (Mulders‐Jones, Mitchison, Girosi, & Hay, 2017) and is linked to an extensive range of emotional and behavioral disorders such as substance abuse (Krug et al., 2009), impulsivity (Boisseau et al., 2012), obsessive–compulsive disorder (Pollack & Forbush, 2013), suicidal attempts (Arcelus, Mitchell, Wales, & Neilsen, 2011), and self‐injurious behaviors (Svirko & Hawton, 2007). Given the impact disordered eating has on an individual's life, it is vital for researchers to identify relevant risk factors to enable the creation of effective prevention and intervention programs. Despite this, the contributing factors of disordered eating are not yet fully understood.

According to the cognitive‐behavioral perspective on body self‐schema (how individuals perceive their body and eating behavior; Greer & Cooper, 2016), an individual's cognitive biases (emanating from their personality traits) can prompt coping and self‐regulating behaviors such as the adoption of dysfunctional eating (Carraça et al., 2011). These cognitive biases are likely to be activated when individuals are excessively focused on food or avoid certain food groups as seen in vegetarianism or veganism (Stein, 1996). That is, an individual's dietary habits may predispose them to disordered eating behavior, where disordered eating can lead to an increasingly restrictive diet (Albery, Michalska, Moss, & Spada, 2019) or conversely, a restrictive diet can lead to disordered eating (Brytek‐Matera, Czepczor‐Bernat, Jurzak, Kornacka, & Kołodziejczyk, 2018). Prior research suggests that a vegetarian or vegan diet may be used by individuals with EDs as a socially justifiable way of restricting their food consumption and controlling their weight, therefore playing a crucial role in disordered eating behavior (Klopp, Heiss, & Smith, 2003; Martins, Pliner, & O'Conner, 1999; Robinson‐O'Brien, Perry, Wall, Story, & Neumark‐Sztainer, 2009; Timko, Hormes, & Chubski, 2012). Furthermore, one study found 52% of individuals with a history of EDs were vegetarian when compared to 12% of individuals within the control group (Barr & Chapman, 2002). While the transition to vegetarianism or veganism is not indicative of an ED, the ongoing graduated restriction of additional food groups is a clear behavioral characteristic of disordered eating (Kadambari, Gowers, & Crisp, 1986).

Although theory (transdiagnostic cognitive‐behavioral model of disordered eating; Fairburn, Cooper, & Shafran, 2003) supports the relationship between diet and disordered eating, research findings on this subject have been inconsistent. For example, studies exploring degrees of dietary restraint among non‐vegetarians and vegetarians found that vegetarians display greater dietary restraint than non‐vegetarians (McLean & Barr, 2003; Robinson‐O'Brien et al., 2009; Trautman, Rau, Wilson, & Walters, 2008), whereas others have found greater dietary restraint in non‐vegetarians (Fatima, Fatima, & Anwar, 2018). While other studies have demonstrated no significant differences between non‐vegetarians and vegetarians (Fisak, Peterson, Tantleff‐Dunn, & Molnar, 2006; Heiss, Coffino, & Hormes, 2017).

A possible explanation for the conflicting results could be the failure of past studies to differentiate between the different levels vegetarianism, often combining them into a single label. Interestingly, studies that have separated vegetarians into the different subgroups have consistently found semi‐vegetarians (Individuals who follow a vegetarian diet but occasionally eat meat or poultry) to display greater dietary restraint then either true‐vegetarians, vegans and omnivores (Curtis & Comer, 2006; Forestell, 2018; Timko et al., 2012). Therefore, it could be speculated that significant results found in previous studies, where participants have not been separated into the subgroups, are likely reflecting the eating attitudes of semi‐vegetarians and might not be characteristic of true‐vegetarians or vegans.

It has also been theorized that vegetarians motivated solely by weight are less inclined to adopt the more restrictive levels of veganism as it requires higher levels of self‐control, commitment, and adoption of a strict lifestyle (e.g., vegans being unable to use leather, suede, and fur; Alvaro, 2017; Curtis & Comer, 2006). This theory has been supported by previous research who found that both non‐vegetarians (Curtis & Comer, 2006) and semi‐vegetarians (Perry, Mcguire, Neumark‐Sztainer, & Story, 2001; Timko et al., 2012) displayed higher levels of dietary restraint than vegetarians or vegans.

While the prevailing theory and empirical evidence support the notion that certain subgroups of vegetarianism are linked to increased risk of disordered eating, little is known about the nature of these associations. For instance, is diet directly related to disordered eating? Or could it be related through intervening variables such as personality traits? Indeed, eating meat has long been a symbol of masculinity and dominance (Rothgerber, 2013; Ruby & Heine, 2011). In contrast, the consumption of fruits, vegetables, and grains generally is associated with femininity, weakness, and selflessness (Beardsworth & Bryman, 1999; Fraser, Welch, Luben, Bingham, & Day, 2000), suggesting that personality factors may play a role in an individual's diet choice.

Selflessness was first proposed as a predisposing personality trait in disordered eating by Goodsitt (1997) who considered EDs as disorders of the self. Self‐psychology posits that ED patients have dysfunctional relationships with their self, thereby deriving their sense of self through food, unwilling to believe that others would willingly fulfill their self‐object needs (Bachar, 1998; Bachar, Gur, Canetti, Berry, & Stein, 2010; Bachar et al., 2002; Bachar, Latzer, Kreitler, & Berry, 1999; Geist, 1989). Therefore, ED patients behave as a selfless person, always fulfilling self‐object needs for others, rather than themselves, resulting in neglect of their basic needs such as food and sustenance (Bachar, 1998).

Bachar et al. (2002) argued that if ED patients did possess traits of selflessness, then they would rate higher than controls on a scale measuring one's rejection of life, that is, the rejection of the self. Indeed, he found that not only did both anorexic and bulimic patients have significantly higher traits of selflessness, they also felt significantly less attraction, and significantly greater repulsion to life than the control group.

More recently, selflessness scores were found to significantly predict disordered eating levels in female adolescents (Pinus, Canetti, Bonne, & Bachar, 2017) and were also found to predict disordered eating behaviors up to four years later (Bachar et al., 2010). These studies lend support to the self‐psychological theory that high selflessness may present as a risk factor in disordered eating, while reduced selflessness may be a protective factor (Bachar et al., 2010).

The primary aim of the current study is to determine whether the personality trait of selflessness mediates the relationships between diet group and disordered eating. If selflessness mediates the relationship between diet group and disordered eating, then it is hypothesized that any relationship between diet group and disordered eating scores will become weaker, or even disappear, once the selflessness scores are controlled for.

## 2 METHOD

### 2.1 Participants

Participants were a nonclinical sample, recruited using a snowballing technique on social media (Facebook), and a first‐year undergraduate sample was recruited through the Charles Sturt University psychology research experience program (SONA). As the current study sought a nonclinical population, participants that answered “yes” to “have you been diagnosed with an eating disorder?” were stopped from proceeding further. The sample included 780 women; however after data cleaning (removal of respondents missing data in questionnaires), a total of 634 women aged between 17 and 68 years (*M* = 28.83, *SD* = 10.71) remained. In the sample of 634 women, 578 (91.2%) self‐identified as Caucasian (non‐Hispanic); 6 (0.9%) identified as Hispanic; 18 (2.8%) identified as Asian; 11 (1.7%) identified as Aboriginal/Torres Strait Islander; 6 (0.9%) identified as Middle Eastern; 2 (0.3%) identified as Pacific Islander; and 2 (0.3%) identified as Black or African American. Eleven (1.7%) respondents selected “Other” classifying themselves as 2 mixed race, 1 Black and Caucasian mix, 4 Caucasian and Asian mix, 1 Eurasian, 2 Maori, and 1 Caucasian, Asian, and Pacific Islander mixed race.

### 2.2 Procedures

Ethics approval was obtained under ethics protocol number H18047 of the Human Research Ethics Committee, at Charles Sturt University (CSU). The quantitative study was delivered online via Qualtrics (provided by CSU School of Psychology). The survey link was distributed via social media and CSU psychology research experience program (SONA). Consent was embedded in the information statement in the survey that had to be electronically agreed to prior to participants being able to continue with the survey questions. Participants were advised that they could withdraw from the study at any point up to final submission. However, because the survey was anonymous, withdrawal after submission was not possible.

Participants were asked to answer demographic questions and then were directed to complete the Selflessness scale (SS; 15 questions) and the EAT‐26 scale (26 questions). The order of the scales (SS, EAT‐26) and the items within the scales were randomized to limit the possibility of order effect and response bias. The duration of the survey was approximately 15 min.

### 2.3 Materials

The online survey comprised of a demographic questionnaire including diet habits, the SS (Bachar et al., 2002) and the EAT‐26 (Garner, Olmsted, Bohr, & Garfinkel, 1982).

### 2.4 Demographics questionnaire

Sex, age, and race/ethnicity were based on self‐report data. Body mass index (BMI; body mass as weight in proportion to height, kg/m^2^) was determined by self‐reported weight and height. Weight status classification was determined based on the World Health Organization (WHO, 1997) standardized categories: BMI < 18.5 = underweight, BMI 18.5–24.99 = normal weight, BMI 25–29.99 overweight, and BMI > 30 = obese. Participant reports of weight trends (gain, loss, or stable) over the past year were collected.

Participants were provided with detailed definitions of all diet categories and then asked to indicate current diet status by responding to the following question “Do you consider yourself a vegetarian or vegan at this time?” (1) yes; (2) no. Participants that answered “yes” were asked to respond to more detailed diet questions (e.g., length of diet), while those that indicated “no” were classified as non‐vegetarians. All participants were then asked to complete a food frequency questionnaire to validate self‐reported diet status.

Using Tonstad, Butler, Yan, and Fraser (2009) dietary classifications, participants were then placed into four categories, making up four levels of the independent variable (non‐vegetarian, semi‐vegetarian, true‐vegetarian, and vegan). Non‐vegetarians were classified as individuals who consumed red meat, fish, poultry, eggs, and dairy more than once a week. Semi‐vegetarians were classified as individuals who ate red meat and poultry less than once a week but more than once a month and still consumed eggs and dairy products (includes semi‐vegetarians and pesco‐vegetarians). Full‐vegetarians were classified as individuals who do not consume red meat, poultry, or fish, but may still consume eggs and dairy products more than once a week (includes lacto–ovo‐vegetarians, lacto‐vegetarians, and ovo‐vegetarians). Vegans were defined as individuals who do not consume any products derived from animals.

### 2.5 EAT‐ 26

The Eating Attitudes Test (Garner & Garfinkel, 1979) was originally designed to detect symptoms of anorexia. Empirical evidence suggests that the EAT‐26 (Garner et al., 1982) can discriminate between healthy controls and individuals with EDs (Garfinkel & Newman, 2001). EAT‐26 scores have also been used to discriminate between individuals with differing levels of disordered eating symptomatology, signifying that the scale is suitable as a continuous measure of disordered eating (Orbitello et al., 2006). The current study therefore used a composite score utilizing the three subscales in the EAT‐26 (Dieting, Bulimia, and Food Preoccupation and Oral Control). In the current study, participants responded to 26 items on a six‐point Likert scale ranging from “*never*” to “*always*.” As this study aimed to explore the entire spectrum of disordered eating in a nonclinical population, each item was scored on a continuum of 1–6 to examine the full range of possible participant responses, with a possible score range of 26–156. High scores suggest increased disordered eating symptomology. Garner et al. (1982) demonstrated reliability and validity of the scale, and Cronbach's α was 0.83. The present study's Cronbach's α was 0.91 suggesting strong internal validity.

### 2.6 Selflessness

The Selflessness scale (SS; Bachar et al., 2002) is a self‐report scale designed to measure an individual's level of selflessness (the proclivity to ignore one's own needs to attend to the needs of others). Participants are asked to score 15 items on a 4‐point Likert scale (1 = *highly disagree*, 2 = *disagree*, 3 = *agree*, 4 = *highly agree*. For the current study, the scale's total had an acceptable albeit low Cronbach's α of 0.68.

### 2.7 Statistical analysis

One participant was identified as being significantly different from the others based on data from the univariate analysis (*Z* score of −3.69 on the SS) and multivariate analysis (Mahalanobis distance of 13.57). This participant was removed leaving a sample of 633.

Assumptions of normality for all scales were assessed for the full sample, and across all levels of the independent variable (diet group) using visual inspections of histograms and box plots. Normality of data was further examined by calculating z scores representing kurtosis and skewness of the scales used for mediation analysis (EAT‐26 scale and SS). *Z* scores above 2.58 were classified as significantly different from normal *p* < .01 (Ghasemi & Zahediasl, 2012).

The EAT‐26 scale revealed both significant skewness 0.86 (*SE *=* *0.20) and significant positive kurtosis 0.71 (*SE *=* *0.20), and therefore, a log transformation was performed on the data. A bootstrapping procedure was utilized for the bivariate correlation and regression analysis to deal with any non‐normality issues in the SS.

All analyses were conducted using SPSS version 25 (IBM, 2017). Descriptive statistics were analyzed for the group as a whole, and for each level of the independent variable (diet group: non‐vegetarian, semi‐vegetarian, true‐vegetarian, vegan). Bivariate correlational analysis was carried out to examine the relationship between diet group, BMI, age, selflessness, and disordered eating.

A mediated regression model was run using PROCESS macro model 4 for SPSS (Hayes, 2017) to test if selflessness mediates the relationship between diet group and disordered eating. Based on past literature findings of significant differences in disordered eating scores between non‐vegetarian, semi‐vegetarian, and vegan diet groups (Curtis & Comer, 2006; Forestell, 2018; Timko et al., 2012), mediation analyses were run using three different reference groups (non‐vegetarian, semi‐vegetarian, and vegan; a true‐vegetarian reference group was not needed as data on true‐vegetarians were subsequently captured while running the three reference groups).

Due to the lack of prior research on the relationship between diet group, selflessness, and disordered eating, the current study's objective was finding unique associations to be tested further (hence, avoiding Type II errors was deemed more critical than making Type I errors). Therefore, Bonferroni corrections were not utilized since these increase the probability of Type II errors and significantly diminish the power of analyses performed (Armstrong, 2014; Perneger, 1998). Consequently, it is imperative to interpret the study's results in the context of the subsequent increased risk of Type I errors.

Hayes (2017) bootstrap method of modeling was utilized to conduct the mediation models and estimate significance. As suggested by Hayes (2017), all mediation models used in this study were subject to 10,000 bootstrap samples, and 95% (percentile) bootstrap confidence intervals were calculated to test statistical inference.

It is important to note that the traditional criteria to establish mediation originally proposed by Baron and Kenny (1986), while historically important, are not consistent with more common practices. More recent assumptions indicate that an individual path in a mediation model need not be significant, and indeed, its significance is not pertinent to whether the indirect effect of a mediation model is significant (see Hayes, 2009, for discussion).

## 3 RESULTS

### 3.1 Dietary group characteristics

Sample characteristics split across diet groups are displayed in Table 1. Vegans reported the highest percentage of weight loss in the previous 12 months, with 26.4% of participants in the vegan diet group reporting weight loss. The semi‐vegetarian group displayed the lowest percentage of weight loss with only 2.8% of participants in the group reported losing weight in the previous 12 months.

**Table 1:** Dietary group characteristics (with outlier)

Group Classification					
	Semi‐Veg	True‐Veg	Vegan	Non‐Veg	
Attributes	*n* (%)/ *M ± SD*	*n* (%)/ *M ± SD*	*n* (%)/ *M ± SD*	*n* (%)/ *M ± SD*	
Age (year)	26.14 ± 8.40	28.88 ± 11.29	27.50 ± 10.10	31.42 ± 11.13	
Height (cm)	166.85 ± 6.41	166.81 ± 6.55	166.26 ± 7.14	165.17 ± 7.76	
Weight (kg)	68.68 ± 18.47	67.87 ± 14.81	65.12 ± 12.01	73.19 ± 17.97	
Characteristics					
Participants	36 (5.7)	104 (16.4)	281 (44.3)	213 (33.6)	
Weight loss	10 (2.8)	28 (7.9)	94 (26.4)	61 (17.1)	
Weight gain	13 (3.7)	25 (7)	58 (16.3)	67 (18.8)	
Length in vegetarian/vegan diet (Years)					
Under 1 year	8 (2)	25 (6.2)	39 (9.7)		
1–2 years	3 (0.7)	24 (6)	72 (18)		
2–5 years	4 (1)	27(6.7)	95(23.7)		
5–10 years	0 (0)	8 (2)	32 (8)		
Over 10 years	2 (0.5)	13 (3.2)	37 (9.2)		
Entire Life	0 (0)	6 (1.5)	6 (1.5)		

Note

Percentages displayed in weight gain/ loss refer to percentages out of *n* = 356. Percentage*s* displayed in “length in vegetarian/vegan diet” refer to percentages out of those who classified themselves as vegetarian/vegan. Semi‐Veg = Semi‐vegetarian, True‐Veg = True‐vegetarian, Non‐Veg = Non‐vegetarian.

Overall, the vegan group contained the highest percentage of “normal weight” individuals, while the non‐vegetarian group contained the highest percentage of “overweight “and “obese” individuals. In addition, the semi‐vegetarian group contained the highest percentage of “underweight” (BMI < 18.5) individuals (Table 2).

**Table 2:** Dietary group frequencies and percentages for BMI categories (with outlier)

Group classification										
BMI category	Semi‐Veg		True‐Veg		Vegan		Non‐Veg		Total	
	*n*	%	*n*	%	*n*	%	*n*	%	*n*	%
Underweight	3	8.3	4	3.9	15	5.4	2	0.9	24	3.8
Normal Weight	22	61.1	65	63.1	191	68.2	100	47.2	378	59.9
Overweight	4	11.1	20	19.4	42	15.0	58	27.4	124	19.7
Obese	7	19.4	14	13.6	32	11.4	52	24.5	105	16.6
Total	36	100	103	100	280	100	212	100	631	100

Abbreviations: BMI, body mass index; Non‐Veg, Non‐vegetarian. % indicates percentage within diet subgroup; Semi‐Veg, Semi‐vegetarian; True‐Veg, True‐vegetarian.

### 3.2 Correlations between selflessness, diet, and disordered eating

To examine the relationship between selflessness, diet, and disordered eating, psychological data obtained from EAT‐26 and SS were analyzed alongside diet and weight data. Pearson *r* Pearson r zero‐order correlations were run for each level of diet group and are presented in table 3. Bootstrapping was utilized using 5,000 repetitions to deal with non‐normality issues. Selflessness scores showed a small significant positive relationship to disordered eating, but only for the vegan group, suggesting that as selflessness levels increased for vegans, so did their level of disordered eating.

**Table 3:** Correlations among study variables across levels of diet group: Age, BMI, EAT‐26‐LOG, and Selflessness

	Diet Group	Age	BMI	Selflessness	EAT‐26‐LOG
Age	Non‐Veg	–	0.251***	−0.118	−0.185*
	Semi‐Veg		0.484**	0.033	−0.304
	True‐Veg		0.158	−0.135	−0.151
	Vegan		0.207***	−0.088	−0.805
		*n*			
BMI	Non‐Veg	*212*	‐	0.162*	0.053
	Semi‐Veg	*36*		0.194	0.104
	True‐Veg	*103*		0.115	0.300**
	Vegan	*279*		0.074	0.060
		*n*	*n*		
Selflessness	Non‐Veg	*210*	*209*	‐	0.130
	Semi‐Veg	*36*	*36*		0.199
	True‐Veg	*104*	*103*		0.102
	Vegan	*276*	*275*		0.142*
		*n*	*n*	*n*	
EAT‐26‐LOG	Non‐Veg	*213*	*212*	*210*	‐
	Semi‐Veg	*36*	*36*	*36*	
	True‐Veg	*104*	*103*	*104*	
	Vegan	*280*	*279*	*276*	

Note.

*p < .05; **p < .01; ***p < .001; two tailed. Bottom triangle denotes sample size *n*.

### 3.3 The mediating effect of selflessness

#### 3.3.1 Comparison against non‐vegetarian reference group

Table 4 illustrates the mediation model between diet group and disordered eating via selflessness with non‐vegetarians as the reference group. Interestingly, vegans had significantly higher selflessness scores (*M* = 42.26, *SE *=* *0.254) than non‐vegetarians (*M* = 41.47, *SE *=* *0.284) and significantly lower disordered eating scores (*M* = 1.78, *SE *=* *0.007) than non‐vegetarians (*M* = 1.81, *SE *=* *0.008).

**Table 4:** Multiple mediation model for diet group, Selflessness and EAT‐26‐LOG (disordered eating). Reference group: Non‐vegetarian

Path	Coeff	*SE*	*t*	*p* value	LLCI	ULCI
*Reference group: Non‐vegetarian*						
*Path a*						
Semi‐vegetarian	0.140	0.766	0.182	.855	−1.37	1.64
True‐vegetarian	0.182	0.509	0.358	.720	−0.818	1.18
Vegan	0.789	0.389	2.03	.043*	0.026	1.55
*Path b*	0.004	0.001	3.41	.001**	0.002	0.006
*Direct effect (C’)*						
Semi‐vegetarian	0.032	0.023	1.41	.158	−0.012	0.075
True‐vegetarian	−0.010	0.015	−0.653	.514	−0.039	0.019
Vegan	−0.032	0.011	−2.85	.004**	−0.055	−0.010
*Total effect (C)*						
Semi‐vegetarian	0.032	0.023	1.43	.155	−0.012	0.076
True‐vegetarian	−0.009	0.015	−0.599	.549	−0.038	0.020
Vegan	−0.029	0.011	−2.56	.011*	−0.052	−0.007

Indirect effect	Effect	Boot SE			BootLLCI	BootULCI
Semi‐vegetarian	0.001	0.004			−0.006	0.008
True‐vegetarian	0.001	0.002			−0.003	0.005
Vegan	0.003*	0.002			0.0001	0.007

Note.

95% Confidence intervals. LLCI = lower limit confidence interval; ULCI = upper limit confidence interval. BootLLCI = bootstrapping lower limit confidence interval. BootULCI = bootstrapping upper limit confidence interval. *SE* = standard error. *p < .05; **p < .01; ***p < .001.

**Figure 1:**
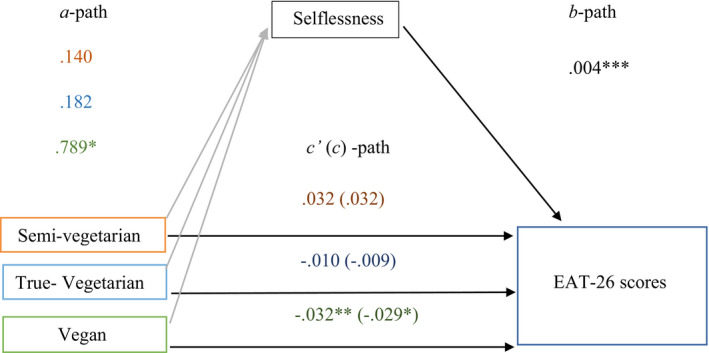
Mediating role of selflessness on the relationship between diet groups and disordered eating with non‐vegetarian as reference group. A‐path: coefficient describing the difference in selflessness in comparison with non‐vegetarian group. b‐path: b‐coefficient reflects change in EAT‐26‐LOG scores per one‐point change in selflessness scores when diet group is constant. c′ (c) ‐path: direct (total) effect. *p < .05; **p < .01; ***p < .001

The vegan diet group was the only group which did not have zero occurs between the upper and lower boundaries of the indirect effect 95% confidence interval, implying that a partial inconsistent mediation had occurred (MacKinnon, Fairchild & Fritz, 2007) and concluding that the mediator variable had acted as a partial suppressor variable. Therefore, the negative relationship between the vegan diet group and disordered eating was strengthened by the addition of selflessness as a mediating variable (Figure 1). (*b* = 0.003, *SE* = 0.002, 95% CI [0.001, 0.007]).

#### 3.3.2 Comparison against vegan, true‐vegetarian, and semi‐vegetarian reference groups

Further analysis was run with the vegan diet group as the reference group. Results indicated that when compared to vegans (*M *=* *1.78*, SE* = 0.007), semi‐vegetarians (*M *=* *1.84, *SE* = 0.025) had significantly higher disordered eating scores, *c* path = 0.061, *t* (622) = 2.78, *p *= .006. No significant difference was found in selflessness scores when semi‐vegetarians and true‐vegetarians were compared to vegans. Contrary to expectations, selflessness did not mediate the relationship between diet group and disordered eating when semi‐vegetarian and true‐vegetarian groups were compared to the vegan diet group, or when the true‐vegetarian and semi‐vegetarian groups were compared to each other.

## 4 DISCUSSION

### 4.1 Diet group and disordered eating

Previous studies examining disordered eating have focused on either personality traits or diet group, with very few exploring both factors simultaneously. The present study therefore sought to answer the following questions: Does selflessness significantly predict disordered eating? and Does selflessness mediate the relationship between diet group (non‐vegetarian, semi‐vegetarian, true‐vegetarian, and vegan) and disordered eating?

In line with previous research (Curtis & Comer, 2006; Timko et al., 2012), the semi‐vegetarian group displayed the highest levels of disordered eating, having higher scores than non‐vegetarians and true‐vegetarians, and significantly higher scores than vegans. Further to this, the current study's results found vegans to have significantly lower disordered eating scores than both semi‐vegetarians and non‐vegetarians. A similar result was found by Timko et al. (2012), who reported lower levels of disordered eating and dietary restraint, in vegans when compared to other diet groups.

The current study's findings raise concerns over past research grouping together semi‐vegetarians and true‐vegetarians (Kadambari et al., 1986; Perry et al., 2001; Trautman et al., 2008), as significant associations between disordered eating and vegetarianism may have simply been artifacts of a larger number of semi‐vegetarians in the study sample. Future research should therefore differentiate between semi‐vegetarians, true‐vegetarians, and vegans when looking at the association between disordered eating and vegetarianism.

The picture that emerges from both previous studies and the present study is that an individual's diet choice and disordered eating behavior appear to be intertwined. The joint link, however, can only be speculated. It is possible that the increased focus on food when an individual commences a semi‐vegetarian diet turns a seemingly innocent diet choice into an obsession and disorder (Lindeman, Stark & Latvala, 2000). However, by this logic, the vegan group, who would require the highest focus on food due to the amount of excluded food groups, should display the highest levels of disordered eating rather than the lowest levels. A more fitting explanation is that the less restrictive levels of vegetarianism, for instance, semi‐vegetarianism, may act as a mask for disordered eating behavior for individuals already suffering from disordered eating, whereas true‐vegetarianism and veganism may represent a lifestyle choice that goes beyond weight and diet preoccupations (Bardone‐Cone et al., 2012; Heiss et al., 2017). However, future research is required for etiological clarification.

### 4.2 Selflessness and disordered eating

In support of the hypothesis, selflessness was found to significantly predict disordered eating. This finding is consistent with previous studies (Bachar et al., 2002, 2010; Pinus et al., 2017) that found selflessness to not only predict disordered eating, but also be a predisposing factor in disordered eating. Another novel finding was that when selflessness was examined by diet subgroup, only the vegan group had a significant positive correlation between selflessness and disordered eating. Moreover, vegans displayed significantly higher selflessness levels than semi‐vegetarians and non‐vegetarians, despite vegans having the lowest levels of disordered eating.

Theory proposes that individuals with EDs derive their sense of self through food and thereby act as a selfless person, always striving to fulfill the needs of others rather than their own (Bachar 1998). From this standpoint, we could reason that vegans have high levels of selflessness as they indirectly derive their sense of self through food by adopting a vegan diet to minimize harm to animals and nature. An alternative explanation could be that when individuals adopt a vegan diet, their levels of selflessness increase as they are exposed to material portraying animal cruelty and environmental impact (Christopher, Bartkowski, & Haverda, 2018), and as selflessness levels increase so does their risk of disordered eating (Bachar et al., 2002).

Yet the question remains, why do vegans who have the highest levels of selflessness also have the lowest levels of disordered eating? As Sarner‐Levin et al. (2018) point out, standing up for one's own beliefs and views may protect individuals against the development of disordered eating, even if they may be predisposed to dysfunctional eating (such as individuals high in selflessness). Additionally, Kessler et al. (2016) found that vegans scored higher on self‐determination than vegetarians and were also less influenced by their social environment than vegetarians. Vegans may then present as a unique group where although they have high levels of selflessness, they also possess strong self‐determination and beliefs, thereby creating a protective factor from disordered eating. However, future research should further investigate the potential protective factors that may be linked to the vegan diet by seeing if the relationship between the vegan diet and disordered eating is mediated by a measure of self‐determination.

### 4.3 The mediating effect of selflessness

Contrary to expectations, the vegan group (compared to non‐vegetarians) was the only group where selflessness acted as an inconsistent mediator by partially suppressing the relationship between diet and disordered eating. This implies that when selflessness was controlled for, vegans displayed substantially less disordered eating pathology than non‐vegetarians. Previous studies have found that a self‐psychological approach (targeting selflessness) significantly reduced ED symptoms in ED patients when compared to cognitive orientation treatment and control/nutritional counseling (Bachar, 2018).

The results from the current study thereby raise the possibility that with targeted intervention on selflessness traits, disordered eating symptoms in vegans may be substantially reduced. Due to the lack of prior research, it remains unclear why no significant associations were found between selflessness and disordered eating in the non‐vegetarian, semi‐vegetarian, or true‐vegetarian group.

## 5 LIMITATIONS

There are several limitations to the current study that should guide the interpretation of results. Firstly, despite ample efforts to recruit semi‐vegetarians, lacto‐vegetarians, ovo‐vegetarians, lacto–ovo‐vegetarians, and pesco‐vegetarians the sample size in these categories was less than optimal, therefore making a comprehensive comparison of all the different levels of vegetarianism unsuitable. Future research should aim to ensure they have adequate sample sizes in each of the diet subgroups to ensure a more detailed comparison can be carried out.

Secondly, the current study is the first study to use the SS in an Australian population. The current study's Cronbach's α for the SS was below the satisfactory level of reliability as suggested by Nunnally (1978). This study highlights the requirement for further validation and testing of the SS cross‐culturally. Further research should aim to either validate the scale using an Australian population or seek to create a scale of selflessness which is suitable for use in a sample of Australian women.

A further concern of the current study was the increased possibility of Type Ⅰ error due to the number of comparisons undertaken to compare non‐vegetarian, semi‐vegetarian, true‐vegetarian, and vegan diet groups. However, even with the increased possibility of Type Ⅰ error, the evident differences in disordered eating between diet groups warrant further investigation. Future researchers should seek to replicate the current study's results using planned comparisons ensuring they had a larger sample of semi‐vegetarians.

## 6 THEORETICAL IMPLICATIONS

The results of the current study raise both theoretical and clinical implications in this field of research. Although the present study indicates that selflessness may be associated with disordered eating; when selflessness is broken down across diet groups, the only group to display a significant association between selflessness and disordered eating was the vegan diet group. The fact that vegans displayed the lowest levels of disordered eating suggests that there may be limitations to be explored in the context of the self‐psychology theoretical framework of EDs (individuals deriving their sense of self through food) when reviewing selflessness across diet groups.

## 7 IMPLICATIONS FOR FUTURE RESEARCH

Inconsistent mediation, as seen in this study, raises concerns for future studies and the possibility of increased Type II error. Inconsistent mediation is characterized by opposing signs on direct effects and indirect effects (negative versus positive) which tend to cancel each other out. Therefore, mediation may be difficult to identify as most models require a significant association between the independent and dependent variables (Baron & Kenny, 1986) which may not appear in a simple regression if the direct effect and indirect effect are canceling each other out. If the current study's findings can be replicated by future research, it may hold implications for future research methodology when exploring the relationship between selflessness and disordered eating.

## 8 CONCLUSION

The aim of this study was to explore the relationship between diet groups, selflessness, and disordered eating. Results showed support of the hypothesis that selflessness is a significant predictor in disordered eating. However, when these relationships were viewed across the different levels of vegetarianism, the results showed that different diet groups displayed unique associations between selflessness and disordered eating. This highlights the need for further research on disordered eating to take into account an individual's diet preference as this may have a significant impact on future findings. Overall, the current study's findings make several contributions to the growing body of literature on disordered eating and serve as a base for future studies exploring the relationship between diet, selflessness, and disordered eating.

## CONFLICT OF INTEREST

The authors declare there is no conflict of interest.

## AUTHOR CONTRIBUTION

M.C conceived and designed the study, collected the cases; analyzed and interpreted the data; and wrote the manuscript. S.Q conceived and designed the study, supervised the project, and edited and contributed to the manuscript. All authors provided revisions and approved the final version of the paper for submission.

### Peer Review

The peer review history for this article is available at https://publons.com/publon/10.1002/brb3.1774.

## Data Availability

The data that support the findings of this study are available from the corresponding author upon reasonable request.
